# Shift in virus composition in honeybees (*Apis mellifera*) following worldwide invasion by the parasitic mite and virus vector *Varroa destructor*

**DOI:** 10.1098/rsos.231529

**Published:** 2024-01-10

**Authors:** Vincent Doublet, Melissa A. Y. Oddie, Fanny Mondet, Eva Forsgren, Bjørn Dahle, Elisabeth Furuseth-Hansen, Geoffrey R. Williams, Lina De Smet, Myrsini E. Natsopoulou, Tomás E. Murray, Emilia Semberg, Orlando Yañez, Dirk C. de Graaf, Yves Le Conte, Peter Neumann, Espen Rimstad, Robert J. Paxton, Joachim R. de Miranda

**Affiliations:** ^1^ Institute of Evolutionary Ecology and Conservation Genomics, University of Ulm, Albert-Einstein-Allee 11, 89081 Ulm, Germany; ^2^ Institute for Biology, Martin Luther University Halle-Wittenberg, Halle (Saale) 061200, Germany; ^3^ Department of Ecology, Swedish University of Agricultural Sciences, Uppsala 750 07, Sweden; ^4^ Norwegian Beekeepers Association, Kløfta 2040, Norway; ^5^ INRAE, UR 406 Abeilles et Environnement, Avignon 84914, France; ^6^ Department of Food Safety and Infection Biology, Norwegian University of Life Sciences, Ås 1432, Norway; ^7^ Institute of Bee Health, Vetsuisse Faculty, University of Bern, Bern 3097, Switzerland; ^8^ Entomology & Plant Pathology, Auburn University, Auburn, AL 36832, USA; ^9^ Department of Biochemistry and Microbiology, Ghent University, Ghent 9000, Belgium; ^10^ German Centre for Integrative Biodiversity Research (iDiv) Halle-Jena-Leipzig, 04103, Germany

**Keywords:** black queen cell virus, deformed wing virus, emerging disease, honeybee, varroa mite, virome

## Abstract

Invasive vectors can induce dramatic changes in disease epidemiology. While viral emergence following geographical range expansion of a vector is well known, the influence a vector can have at the level of the host's pathobiome is less well understood. Taking advantage of the formerly heterogeneous spatial distribution of the ectoparasitic mite *Varroa destructor* that acts as potent virus vector among honeybees *Apis mellifera*, we investigated the impact of its recent global spread on the viral community of honeybees in a retrospective study of historical samples. We hypothesized that the vector has had an effect on the epidemiology of several bee viruses, potentially altering their transmissibility and/or virulence, and consequently their prevalence, abundance, or both. To test this, we quantified the prevalence and loads of 14 viruses from honeybee samples collected in mite-free and mite-infested populations in four independent geographical regions. The presence of the mite dramatically increased the prevalence and load of deformed wing virus, a cause of unsustainably high colony losses. In addition, several other viruses became more prevalent or were found at higher load in mite-infested areas, including viruses not known to be actively varroa-transmitted, but which may increase opportunistically in varroa-parasitized bees.

## 1. Background

Biological introductions and invasions of non-native species have rapidly increased in recent decades as hallmarks of the Anthropocene: a consequence of increasing international trade, globalization and climate change [1]. These invasions often result in important changes to species communities, biodiversity and ecosystem services through mechanisms such as species competitive exclusion [2] and the emergence of disease, with direct effects on human health, food security and wildlife conservation [3,4]. One particularly worrisome class of invaders are disease vectors, such as biting ticks, mites and phytophagous insects (e.g. aphids, thrips, whiteflies and mealy bugs). These parasites often harbour piercing mouthparts used to suck nutrients (blood or sap) from their hosts, and by so doing they transmit microbial diseases such as viruses or bacteria.

Such invasive vectors can spread disease over large geographical scales into new host populations or species, and are therefore referred to as disease facilitators [5]. Prominent examples are the emergence in the early 2000s of zoonotic viral diseases like dengue and Zika [6] as a direct consequence of the rapid geographical range expansion of mosquito vectors [7,8], or the expansion of cassava mosaic disease (CMD) viruses in Eastern and Central Africa following outbreaks of vector whitefly populations, with important agricultural and economic consequences [9]. The acquisition of a novel vector-borne transmission route by endemic pathogens following a vector invasion is also expected to markedly alter its epidemiology through changes in transmission and virulence, potentially resulting in higher prevalence and infection intensity [10].

In honeybees, the global dispersal of the ectoparasitic mite *Varroa destructor* [11] is another good example of an invasive vector leading to disease emergence, and one of the most ubiquitous and serious threats for the beekeeping and pollination industries [12–14]. *Varroa destructor*'s (henceforth ‘varroa mite’) original host was the Asian honeybee (*Apis cerana*), but switched host to the Western honeybee (*Apis mellifera*), after *A. mellifera* had been introduced to East and South Asia from its native range in Europe, the Middle East and Africa [11]. Varroa subsequently spread globally as a result of human-mediated long-distance movement of bees, primarily through the global trade in honeybee (*A. mellifera*) queens and adult bee ‘starter packages’ [11]. As a consequence, varroa mites are currently found on all major land masses harbouring *A. mellifera*, including most recently Australia despite the country's strict quarantine regulations [15]. In fact, human-mediated honeybee transport, whether deliberate or accidental, legal or illegal, has been the cause of nearly every breach of varroa quarantine throughout its short history, including in the UK [16], Hawaii [17], New Zealand [18], the Azores [19], Madagascar [20], Mauritius [21], and recently the Åland Islands [22] and Ushant, an island off the west coast of France [23]. Only a few islands, remote valleys and latitudes beyond the natural range of honeybees [24] remain free of varroa, while varroa eradication programmes are also occasionally successful for small, isolated islands with a limited and well-monitored bee population [25].

Varroa mites feed on the fat body and haemolymph of pupal and adult honeybees by sucking host tissue through the host's exoskeleton [26]. While feeding on its host, the mite may transmit viral diseases. The most prominent viral disease of honeybees is deformed wing virus (DWV), which, in conjunction with varroa, is the main driver of overwinter worker mortality and colony failure in *A. mellifera* [27–32], and currently represents a pandemic [33]. Following the global dispersal of varroa, DWV rapidly switched from being a primarily orally and sexually transmitted, incidental, low-abundance and asymptomatic virus into a highly prevalent, high-abundance (load), vector-transmitted virus with severe symptoms at individual and colony levels [34–36]. Its increased virulence when injected into a host by the mite is probably the result of circumventing physical and biological barriers between tissues [37], potentially coupled with host immune-suppression by the mite [38–41] and avoidance of social hygienic vigilance by adult bees [42,43]. In recent years, the global establishment of varroa probably prompted the selection of an emerging variant of DWV, genotype B, capable of replicating within the mite, which is swiftly replacing the previously dominant variant, DWV genotype A [44–46].

Increased virulence upon newly acquired vector-borne transmission is generally expected, either as a dose-dependent effect or through a pathogen's adaptation to the vector [38,47,48]. Thus, beyond the DWV pandemic, the invasion of varroa mites has probably impacted the epidemiology of other viruses in honeybees [18,49]. While early bee virology identified a set of diseases that may be transmitted by varroa [50], changes in virus epidemiology following mite invasion of populations of *A. mellifera* remain unclear. Several studies have locally identified viruses that have higher prevalence in varroa-infested population compared with varroa-free colonies, such as Kashmir bee virus (KBV) and sacbrood virus (SBV) in New Zealand [18], or black queen cell virus (BQCV) and slow bee paralysis virus (SBPV) in Europe [51]. By contrast, none of these viruses seem to have been impacted by the arrival of the mite in Hawaii [17], illustrating the need to synthesize the role of varroa in shaping the honeybee viral landscape. At the virome level, two studies, from Hawaii [52] and the Channel Islands [51], confirmed the general trend of increased DWV loads in varroa-infested honeybees, but did not detect a trend for any other virus.

Gaining a better understanding of pathogen epidemiology in response to vector-borne transmission in natural populations, beyond well-reported pathogenic diseases such as DWV in honeybees, is key for the control and mitigation of disease emergence. Here, we hypothesized that varroa mites have an effect on the epidemiology of several other viruses*.* To test this, we analysed historical samples of adult *A. mellifera* collected during the early 2010s in front and behind the varroa expansion front in four independent geographical regions of the world, for the prevalence and loads of 14 viruses. Using current knowledge on the major transmission routes of different bee viruses [35] and their virulence, we expected viruses known to be varroa-transmitted but with low to moderate virulence, such as DWV, to show increased prevalence and load in the presence of the mite, and varroa-transmitted viruses with high virulence, namely acute bee paralysis virus (ABPV), KBV and Israeli paralysis virus (IAPV) to drop in prevalence [48,49,53]. By contrast, for those viruses not actively transmitted by varroa, we expected no major effect of the presence of the mite on either virus prevalence or loads, or an indirect and moderate increase due to the debilitating effects of varroa and its transmitted viruses on the general health and immune competence of the adult bee hosts.

## 2. Methods

### 2.1. Experimental design

To determine the impact of varroa invasion on the virus assemblage of honeybee populations, we analysed historical samples collected from four independent geographical regions: Canada, the United Kingdom and the Isle of Man (henceforth British Isles), New Zealand and Scandinavia (Norway and Sweden). In each region, the honeybee samples were collected from areas determined by active mite infestation surveys to be varroa-free or varroa-infested at the time and location of sampling, using several mite infestation detection methods [54]. Areas considered varroa-free are islands and valleys where varroa mites were not yet reported in or before the year of sample collection, despite beekeeper awareness and regular monitoring for mite presence. However, many of these sites will now (in 2024) no longer be varroa-free, more than ten years after these samples were collected. Analysing historical samples from four independent regions of the world enabled us to detect general patterns in the viral community's response to varroa invasion, independent of any local geographical and bee-virus idiosyncrasies.

**Figure 1.**
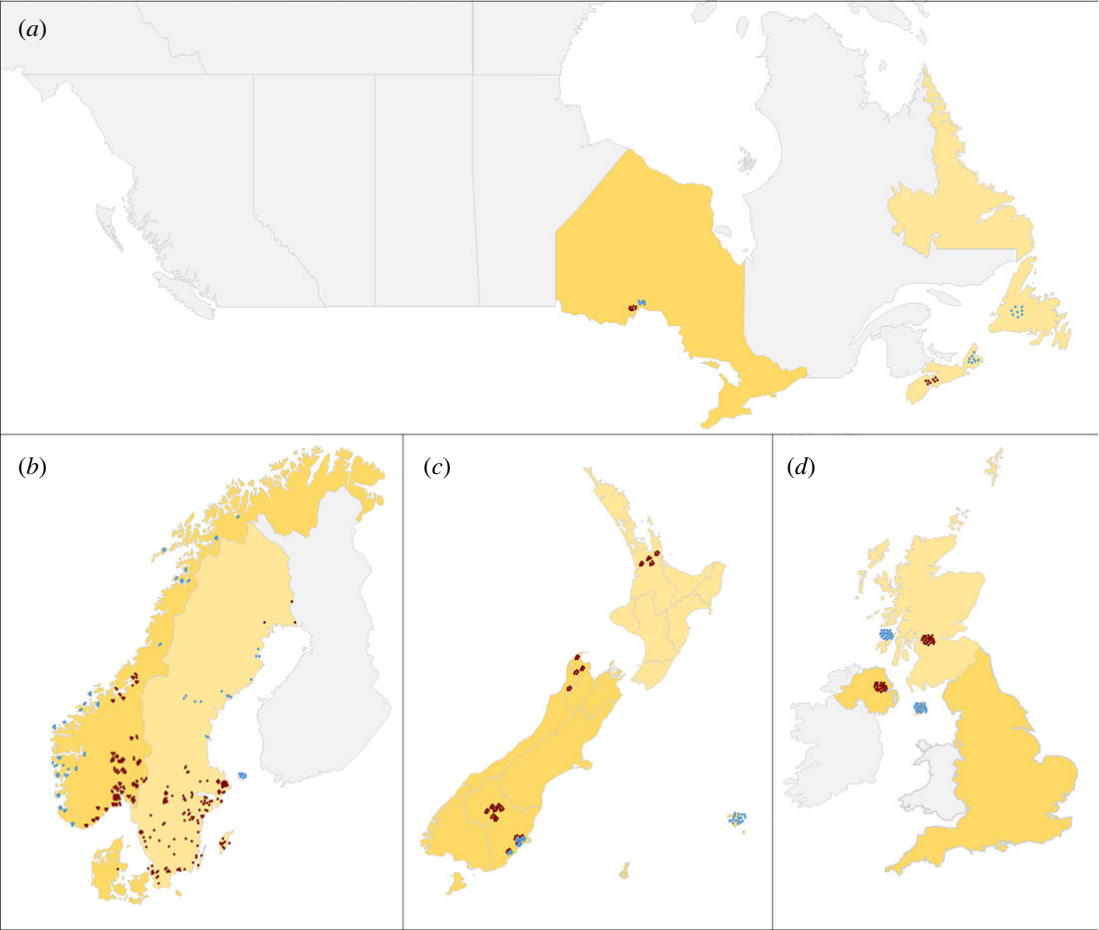
Sampling sites in geographical regions (*a*) Canada, (*b*) Scandinavia, (*c*) New Zealand and (*d*) British Isles. Each point represents a sampled hive, where blue dots represent hives from varroa-free areas, and red dots hives from varroa-infested areas at the time of sampling. In New Zealand, overlapping blue and red dots show the dynamic movement of the varroa expansion front during the sampling period.

Adult worker honeybees were sampled inside hives from a total of 654 colonies, with a third of these (216) located in varroa-free areas (figure 1). We measured the presence and abundance of 14 RNA viruses in *A. mellifera* by quantitative RT-PCR. For this screening, we selected nine well-known viruses that cause pathological damage to either adult bees or brood [35,36,55]: acute bee paralysis virus (ABPV), black queen cell virus (BQCV), chronic bee paralysis virus (CBPV), deformed wing virus genotype A (DWV-A), and genotype B (DWV-B; formerly *Varroa destructor* virus-1, or VDV-1) [34,56], Israeli acute paralysis virus (IAPV), Kashmir bee virus (KBV), slow bee paralysis virus (SBPV) and sacbrood virus (SBV). We surveyed five additional viruses that were more recently characterized and for which we have only limited knowledge of their pathologies in bees: Lake Sinai virus strains 1 and 2 (LSV-1, LSV-2) [57], aphid lethal paralysis virus (ALPV) [58], Big Sioux River virus (BSRV) [58] and bee macula-like virus (BeeMLV, formerly known as *Varroa destructor* macula-like virus, or VdMLV), which is a virus that has previously been associated with *V. destructor* [59].

### 2.2. Sample management

Samples were collected during the summer–autumn of the 2010–2013 bee seasons, and before autumn treatment against varroa. Depending on availability, 1 to 30 colonies were sampled from the same beekeeper. Slightly different sampling strategies were applied in the different territories. In Canada and the British Isles, the sampling strategy was based largely around beekeeping operations, with many samples supplied by relatively few beekeepers and similar numbers of samples on either side of the varroa front. In Norway and New Zealand, the samples were collected by apiary, each apiary contributing a uniform number of samples (three–four in Norway, five in New Zealand), while in Sweden most of the samples were selected on geographical coverage, with mostly single colonies from each location (figure 1). The New Zealand and Scandinavian samples were transferred to −80°C within 24 h of collection; see Mondet *et al*. [18] for more description of New Zealand samples. The Canadian and British Isles samples were crushed in RNAlater (Qiagen, Hilden, Germany) and shipped on ice for a maximum of 72 h to the analysing laboratory, where they were stored at −80°C until processing [60]. Because samples were independently screened, not all viruses were surveyed in all regions. Six viruses were quantified in all samples: ABPV, BQCV, CBPV, DWV-A, IAPV and SBV. KBV was not tested in all samples from Scandinavia. SBPV, LSV-1 and LSV-2 were not tested in samples from New Zealand, while DWV-B was tested in only two regions (Canada and the British Isles). ALPV, BSRV and BeeMLV were only screened in the Scandinavian samples (see electronic supplementary material, table S1).

### 2.3. Sample processing

We followed standardized methods to isolate RNA from bee samples [61,62]. Pools of 30 adult bees were pulverized in mesh bags (BioReba AG, Reinach, Switzerland) with a pestle and liquid nitrogen and homogenized in 6 ml sterile water. Total RNA was extracted from 100 µl of this homogenate using the Plant RNeasy kit (Qiagen, Hilden, Germany) following the manufacturer's instructions, eluting in a final volume of 50 µl sterile water.

### 2.4. Two-step reverse transcription–quantitative polymerase chain reaction assays

Approximately 1 µg RNA from each sample was converted with random hexamer primers to cDNA using a Moloney murine leukemia virus (M-MLV)-based first strand cDNA synthesis kit (Promega, Madison, WI, USA) following the manufacturer's recommendations. The cDNA was diluted 10-fold in sterile water and stored at −80°C until further use as template in a range of quantitative polymerase chain reaction (qPCR) assays for quantifying the absolute loads (in genome equivalents) of each virus per bee (electronic supplementary material, table S2), using a calibration curve consisting of a 10-fold dilution series of external quantification standards of known concentration for each virus assay [61,63]. Similar assays were also run for the absolute quantification of the mRNA levels of two internal honeybee reference genes (β-actin and RP49) in each sample (electronic supplementary material, table S2). The qPCR assays were run in duplicate in 20 µl volumes with SYBR-Green qPCR buffer (BioRad, Hercules, CA, USA) containing 3 µl template (either diluted sample cDNA, negative template-free controls or positive controls from the 10-fold quantification dilution series) and 0.2 µM each of the forward and reverse assay primers with the following amplification profile: 5 min at 95°C, followed by 40 cycles of (10 s at 95°C—30 s at 57°C—read), followed by a melting curve analysis for ascertaining product specificity (1 min at 95°C; 1 min at 55°C, then 0.5°C s^−1^ to 95°C). Each 96-well PCR plate was dedicated to just a single target (virus or internal reference gene), with 16 wells dedicated to duplicates of the positive and negative controls, and the remaining wells dedicated to biological samples.

The result of each reaction was first evaluated using their melting curve profile to confirm the identity of the PCR product. Any conflict between duplicate results (e.g. replicate cycle quantifications (Cqs) differed by greater than 1 replication cycle) were resolved by repeating the assay. The mean Cqs of the duplicate runs were converted to absolute levels of each virus/reference gene in each sample, using the external calibration standard curves for the different assays. The absolute levels of the internal reference genes were subsequently used to normalize the quantitative virus data so as to account for sample-specific differences in RNA quality and quantity [61–63]. These normalized absolute levels of the viruses were then used as starting data for any subsequent analyses. This includes both categorical data (presence or absence, for analyses of virus prevalence) and quantitative data (for analyses of the load of each virus in each positive sample). The genetic identity of the PCR products for DWV-B from Canada was confirmed by Sanger sequencing, using local commercial sequence service providers, and has the GenBank accession number: OR530177.

### 2.5. Statistical analyses

We calculated true prevalence (with 95% confidence intervals) using the R package *epiR* [64] and the function epi.prev(), to account for assay efficiency and sensitivity, which was conservatively set at 95% [65]. Virus prevalence was analysed with generalized linear mixed models (GLMM) using the R package *lme4* [66], with the presence of varroa as an explanatory variable, geographical region and beekeeper ID nested within geographical region as random variables, a binomial error distribution and logit link function. Region was omitted from models when the virus was surveyed or found in one region only (*n* = 2 viruses) and beekeeper ID was omitted when models did not converge (*n* = 3 viruses). To ensure geographical region as random factor was not hampering model validity, we ran another set of linear models where region was a fixed effect (electronic supplementary material, Models M1), which delivered qualitatively the same overall results.

We used another GLMM model with a Poisson error distribution to test the effect of varroa presence on the number of virus species per sample, restricting this analysis to the six viruses surveyed in all colonies: ABPV, BQCV, CBPV, DWV-A, IAPV and SBV.

Virus loads were log_10_ transformed prior to analysis, both for statistical reasons and because pathogens like viruses have an inherent capacity for exponential growth, and their amounts in the host are typically distributed on a log-linear scale [62]. We analysed viral loads using linear mixed models, with varroa presence as explanatory variable and geographical region as random variable for viruses detected in more than one region, and linear models for viruses detected in only one region. All models were checked for overdispersion using the overdisp_fun() function. We applied a false discovery rate (fdr) *p*-value adjustment to account for multiple testing. Note that statistical models were not run for ALPV, BeeMLV, DWV-B and SBPV as there were too few positive samples for these viruses across the dataset. Because of the large number of zero values in our dataset, we also ran additional models in which we fitted our data with zero-inflated models (electronic supplementary material, Models M2), which delivered qualitatively the same overall results.

To identify major changes in viral titres that accompanied the presence of varroa, we performed a principal component analysis (PCA) using log_10_-transformed centred and scaled loads of the seven most surveyed viruses (ABPV, BQCV, CBPV, DWV-A, IAPV, KBV and SBV) and the presence/absence of varroa in the sampling site. PCAs were built using the function prcomp(): individual sample points from PCAs were projected over three dimensions, which explained most of the data variation, with ellipses representing the area including 75% of the data points around the centroids. Variables contributing significantly to the principal component (PC) axes were determined using the *PCAtest* package [67] with 100 random permutations and bootstrap replication, and an alpha threshold of 0.05.

## 3. Results

### 3.1. Virus prevalence

**Figure 2.**
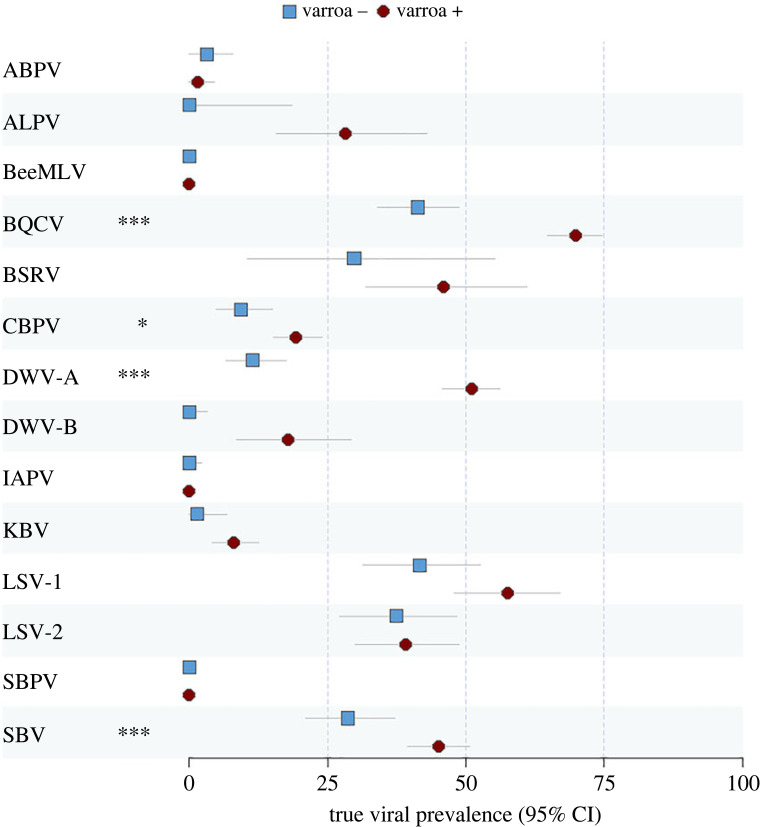
Estimated true prevalence for all 14 viruses (and estimated 95% confidence intervals), comparing *A. mellifera* colonies from varroa-free areas (blue squares) with varroa-infested areas (red circles). Models outcome and sample size are shown in electronic supplementary material, table S3. The fdr adjusted *p*-value for the level of statistical confidence regarding the difference between the varroa-infested and non-infested virus prevalence is indicated as follows: * *p* < 0.05, *** *p* < 0.001.

Across all sites and varroa status, the highest viral prevalence was found for BQCV (estimated true prevalence: 60.36%, 0.95 confidence intervals (CI) 56.12–64.55, *N* = 654) and LSV-1 (50.47%, CI 43.32–57.50, *N* = 238) while BeeMLV was not detected in any sample (*N* = 250 from Scandinavia) (figure 2). The emerging DWV-B, tested only on samples from the British Isles and Canada, was found at lower prevalence than variant DWV-A (DWV-A: 37.97%, CI 33.84–42.18, *N* = 654; DWV-B: 14.58%, CI 8.44–22.07, *N* = 160). Only one case of DWV-B was found in Canada, which was confirmed by Sanger sequencing the PCR product (GenBank accession number: OR530177).

In models examining the effects of varroa on virus prevalence, mite presence was significantly associated with higher prevalence of BQCV, CBPV, DWV-A and SBV after fdr *p*-value correction (electronic supplementary material, table S3). When testing the effect of varroa presence on the number of co-occurring viruses among the six viral species present in all regions, we found colonies from varroa-infested areas harbouring a greater number of viruses than varroa-free colonies (varroa-infested mean (s.e.m.) = 1.90 ± 0.06, varroa-free = 1.05 ± 0.08 viruses; GLMM varroa effect Z = 5.758, *p* < 0.001).

### 3.2. Virus loads

**Figure 3.**
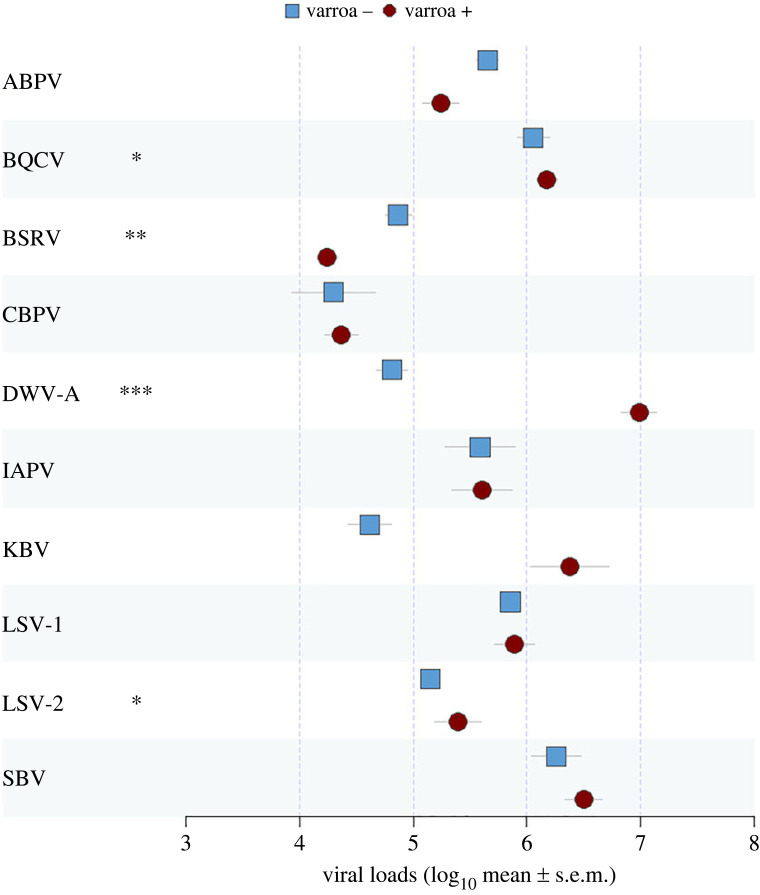
Viral loads expressed as mean log_10_-transformed viral genome equivalents per worker bee (± s.e.m.) for 10 out of 14 tested viruses, comparing *A. mellifera* colonies from varroa-free areas (blue squares) with varroa-infested areas (red circles). ALPV, BeeMLV, DWV-B and SBPV are not shown as too few samples were positive. Models outcome and sample size are shown in electronic supplementary material, table S4. The fdr adjusted *p*-value for the level of statistical confidence regarding the difference between the varroa-infested and non-infested virus loads is indicated as follows: * *p* < 0.05, ** *p* < 0.01, *** *p* < 0.001.

The highest viral loads per honeybee worker in varroa-free populations were for BQCV and SBV (median and interquartiles: BQCV = 7.72 × 10^5^, Q1 = 1.79 × 10^5^, Q3 = 5.86 × 10^6^; SBV = 5.13 × 10^5^, Q1 = 2.44 × 10^5^, Q3 = 4.49 × 10^6^) while in varroa-infested populations, the highest viral loads were observed for DWV-A (1.92 × 10^6^, Q1 = 1.83 × 10^5^, Q3 = 2.34 × 10^8^) (figure 3). In models examining the effects of varroa on viral loads, while controlling for geographical region as a random effect, mite presence was significantly (after fdr *p*-value correction) associated with higher loads of BQCV, DWV-A and LSV-2 (electronic supplementary material, table S4), and lower loads of BSRV. Although higher KBV loads were found in mite-infested areas, this difference was not significant, probably due to the low number of positive samples (10) from mite-free areas.

### 3.3. Viral community

**Figure 4.**
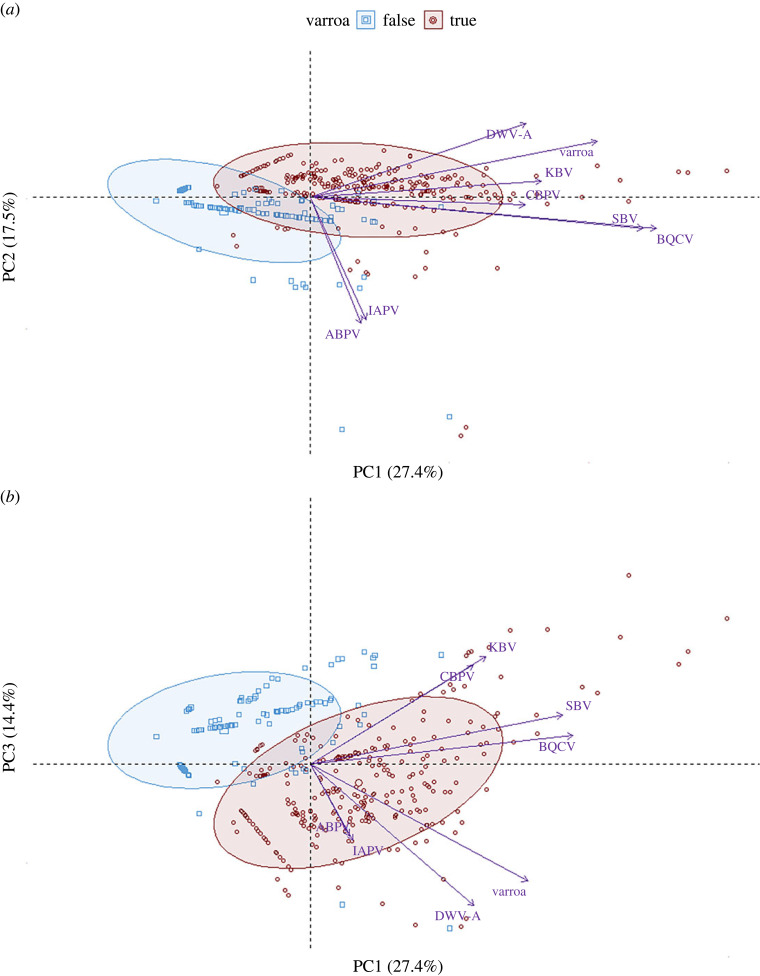
Scatterplots of viral loads analysed by PCA, and projected along the three principal components ((*a*): PC1 and PC2 (*b*): PC1 and PC3). Blue squares represent colonies from varroa-free areas, while red circles represent colonies from varroa-infested areas. Ellipses represent the area including 75% of the data points around the centroids (larger data points).

Analysis of loads from the seven most common viruses by PCA revealed significant *p*-values for *Psi* (2.3852, maximum null = 0.2469, minimum null = 0.0565, *p*-value < 0.05) and *Phi* (0.2064, maximum null = 0.0664, minimum null = 0.0318, *p*-value < 0.05) compared with null values, indicating a non-random correlational structure in the data. The first three components of the PCA explained 59.33% of the variance in the data and all contributed to a significant portion of variation when compared with null models generated by permutation (electronic supplementary material, table S5), with PC1 (27.4%) accounting for about the same amount of variance as PC2 (17.5%) and PC3 (14.4%) combined. The statistically significant variables contributing to PC1 are, in order of effect size, BQCV load, SBV load, the presence of varroa, DWV-A load and KBV load, with all effects pointing towards an increase along the abscissa (figure 4*a*). ABPV and IAPV loads contributed significantly to PC2, with the effects practically co-aligned in size and direction, but orthogonal to the presence/absence of varroa (figure 4*a*). DWV titres contributed significantly to PC3 and to the separation between varroa-infested and varroa-free sites (figure 4*b*), while furthermore also separating out DWV-A and varroa-status from the other viruses along PC1. Both PC1 and PC3 contributed to the separation of samples from varroa-free and varroa-infested areas, along both the abscissa and ordinate axes (figure 4*b*), but not PC2 (figure 4*a*). Scatterplots showing the geographical origin of samples do not show a clear separation of data points by region (electronic supplementary material, figure S1).

## 4. Discussion

Invasive pathogen vectors are key factors in disease emergence and re-emergence, by promoting pathogen transmission efficiency and/or infection intensity. These mechanisms are often well described for prominent and symptomatic diseases, but vectors may probably have a wider effect on their hosts' pathobiome. Taking advantage of the dynamic distribution of *V. destructor*, a newly acquired invasive parasitic mite and virus vector, we investigated the impact of its recent dispersal into previously uninfested territories on the virome of its host, the Western honeybee *A. mellifera*. We hypothesized that varroa has affected the epidemiology of several bee viruses, in addition to the emergence of DWV as the best adapted virus to varroa-mediated transmission [48] and therefore currently the most serious viral disease of *A. mellifera*.

As expected, the presence of the varroa mite dramatically increased the prevalence and load of DWV-A, and this effect was by far the most important change in the honeybee viral community in 2010–2013. In addition, several other viruses have also become more prevalent in presence of the mite, such as BQCV, CBPV and SBV, or were found at higher loads in mite-infested areas, like BQCV and LSV-2. Conversely, one virus, BSRV, was found at lower loads where varroa was present. The emergent DWV-B virus strain, which has become increasingly dominant worldwide during the past decade [44–46], was not quite as prominent in the early 2010s when our samples were collected, although it was already present in the British Isles and Canada. Two members of the highly virulent ABPV complex (ABPV and IAPV) were not associated with varroa parasitism, despite conclusive evidence that these viruses can be actively transmitted by varroa [35]. Historically, these ABPV-complex viruses are generally the first viruses to be associated with varroa, both in prevalence and loads [18,49,53], before being rapidly superseded by DWV on account of their excessive virulence at individual [43] and colony [49,53] level. This temporal context for the varroa–ABPV-virus complex relationship may have disrupted any statistical association between these viruses and geographical varroa presence in our study. Overall, we found that recent varroa mite invasion altered the honeybee virus composition by increasing the prevalence and loads of several viruses, independent of geographical region. We found honeybees in mite-infested areas to carry significantly more virus species and a greater viral load than honeybees from varroa-free populations, illustrating how a vector, *V. destructor* in this case, can modify the viral landscape of its host.

DWV-A displayed the most dramatic epidemiological shift in response to invasion by varroa. In our samples, this variant was more prevalent and at much higher load in honeybees from varroa-infested territory than in nearby varroa-free territory. This confirms similar findings from local field surveys in Hawaii [17,52] and the Channel Islands [68], where varroa-free colonies were also still present at the time of sampling. Experimental work has demonstrated that the new route of transmission provided by the mite—the direct injection of the virus into honeybee pupae instead of oral transmission in adults, coupled with an immune-suppressive effect of the mite [38]—has promoted very high viral loads and the rapid spread of the disease [69–73]. In the two regions where we surveyed for DWV-B, Canada and the British Isles, this emerging DWV variant was still only rarely detected, relative to DWV-A, when these samples were collected in 2010–2013. However, through this one positive sample from Canada, we can trace the arrival of DWV-B in North America to the early 2010s, similar to that of another retrospective study of historical samples [74]. This genotype is now much more prevalent worldwide (e.g. [30,75]) and is apparently currently replacing DWV-A in varroa-infested honeybee populations [44–46], underscoring the continuing influence of the mite on bee virus epidemiology and evolution. The ability of DWV-B to replicate in the mite [76], in contrast to DWV-A, which seems to be primarily mechanically transmitted by varroa ([77], but see [78]), along with the faster replication of DWV-B in honeybees [79], might have contributed to the progressive displacement of DWV-A by DWV-B. Other factors may have a role in the competitive advantage of DWV-B against DWV-A, such as a higher viral load in adult bees [72] but a similar virulence in pupae [71,80], contributing to its rapid spread by varroa.

Beyond the effect on DWV, we hypothesized that other viruses may probably have experienced altered epidemiology following the global dispersal of varroa mites. Indeed, we identified new possible varroa–virus associations. For instance, BQCV, the most widespread and prevalent virus in honeybees [36], also increased in prevalence and load in the presence of the mite. A lower BQCV prevalence in varroa-free honeybee populations in western Europe has previously been reported [51], although this was never formally associated with (lack of) varroa transmission. Whether BQCV can be successfully vectored or passively transmitted by the mite remains to be determined experimentally. Although replication intermediates of BQCV have been detected in varroa [81], these can just as easily have come from the bee tissues that the mite was feeding on and, in surveys, BQCV is only seldomly detected in mites [78]. Experimental injection of BQCV in honeybee pupae, mimicking varroa-mediated transmission, suggests that BQCV may be too virulent to develop an epidemically stable and successful virus-vector relationship with varroa at the colony and inter-colony levels [82,83]. Other factors, such as infection dose delivered by the mite, may be important to determine the nature of the association between the vector and the virus [84]. The apparent rise in prevalence and load of BQCV in presence of varroa mites might well be due to the suppressed immune response of bees as a consequence of varroa parasitism or infection by other viruses such as DWV [38,85].

Two other viruses, CBPV and SBV, were also found at higher prevalence in honeybee samples from varroa-infested areas. However, in contrast to DWV and BQCV, these were not associated with increased viral loads, suggesting that these viruses may be opportunists, benefitting indirectly from with the general debilitation of honeybees by the mite rather than being actively transmitted. Although CBPV and SBV were occasionally reported in varroa [78,81,86], neither of these viruses have been conclusively demonstrated to be transmitted by the mite [35]. Other mechanisms may lead to greater prevalence by inter-colony spread. Horizontal hive-to-hive transmission may result from the drifting of foragers (i.e. bees entering the wrong colony), or from nest robbing [87,88]. Although honeybee colonies have guards at the hive entrance, acceptance of drifting workers is more common in varroa-infested colonies [89] or diseased hives [90], making them recipients to new infections [91]. This possible facilitation of virus spread by varroa could represent a significant health issue as CBPV and SBV may induce significant losses [55].

The presence of varroa significantly changed viral loads of two other viruses: LSV-2, showing higher loads, and BSRV showing lower loads in honeybees from varroa-infested regions. LSV-2 has never been linked to varroa transmission and is rarely found in mites [57,81,92], but its presence has been correlated with poor colony health [93,94]. We hypothesize that increased levels of LSV-2 may be an opportunistic response to immune-suppressed honeybees infested with mites and infected with other viruses such as DWV [38,39,85]. Increased loads of LSV-2 may also have been exacerbated by the indirect effect of miticide treatments widely used to control varroa infestation levels, which reduce honeybee immunocompetence and increase viral levels [95]. These general health and immunity-related factors can of course also affect the loads of the varroa-transmitted viruses (DWV, ABPV) and the less easily transmitted or indirectly associated viruses (e.g. BQCV, CPBV, SBV). By contrast, BSRV showed lower loads in colonies from varroa-infested regions. Although commonly found in surveys [58,96,97], BSRV has never raised any concern for beekeepers and its effect on honeybee health is unknown. The apparent negative association with the mite might explain why this virus stayed under the radar until the development of modern molecular techniques. We speculate that the presence of BSRV, which is phylogenetically closely related to the aphid-infecting *Rhopalosiphum padi* virus [98] and is frequently detected in a range of aphid species [99,100], is an incidental virus in honeybees, acquired passively when honeybees feed on honeydew collected from aphids, particularly when or where floral sources of nectar are lacking [62]. This would confine BSRV, as well as ALPV, another aphid infecting virus found in bees [97,98], to the bee gut and away from the tissues accessed by varroa for feeding [26].

## 5. Conclusion

The distribution of varroa mites, with multiple independent varroa-free zones, offered a unique opportunity to quantify the effect of an invasive vector on its host viral communities. Our results support the prediction of increased prevalence and load of several viral pathogens upon their shift to vector-borne transmission. This illustrates the importance of monitoring the geographical and host range expansion of disease vectors in human populations, as well as in domestic animals, wildlife and crops. Vector invasion is probably the most important source of disease emergence, and climate change may exacerbate this phenomenon [101], including in communities of bees [102,103].

Here we showed how varroa mite invasion has altered the viral community of honeybees, with several viruses showing increased prevalence or load, or both in the case of DWV and BQCV. Beyond the burden that both parasites and viruses represent for honeybee health, increased transmission potential of viruses is a serious threat to other insect pollinators. More than 20 000 species of wild bees play critical roles in agriculture and native ecosystems [104]. Increased viral loads in honeybees in the presence of varroa may lead to undesired spillover to co-foraging insect pollinators or bee predators [51,68,75,105,106], the consequences of which for biodiversity and ecosystem functioning deserve more attention [37], as do the risks of developing new reservoirs or outbreaks of novel variants [107]. The development of a global beekeeping industry that includes current strategies of varroa mite control or the development of mite-tolerant honeybees [108,109] should include awareness of multi-host virus dynamics and strategies that mitigate the risk of viral spillover from honeybees to wild bee species and other flower visitors [37,110].

## Data Availability

The original data and supporting results are publicly available on FigShare, under doi:10.6084/m9.figshare.24083637 [111]. One genetic sequence has been deposited on GenBank, accession number: OR530177. Supplementary material is available online [112].
